# Physical Activity for Anxiety for Autistic People: A Systematic Review

**DOI:** 10.1007/s10803-024-06356-9

**Published:** 2024-05-16

**Authors:** Kathryn Riis, Brittany Samulski, Kristina A. Neely, Patricia Laverdure

**Affiliations:** 1 Department of Kinesiology, Auburn University, 301 Wire Rd, Auburn, AL 36830 USA; 2 Department of Kinesiology and Rehabilitation, Old Dominion University, Norfolk, VA USA; 3 Department of Rehabilitation Sciences, Old Dominion University, Norfolk, VA USA

**Keywords:** Autism, Physical activity, Anxiety, Systematic review

## Abstract

Clinical anxiety is a common comorbidity in autistic people. Due to the prevalence of anxiety in the autism population and the adverse effects it causes, there is a critical need to develop effective interventions which address anxiety symptoms for autistic people. Therefore, the purpose of this systematic review was to examine the effectiveness of the use of physical activity as an intervention to reduce anxiety in autistic people. Three databases PubMed, PsychInfo, and Cochrane RCTs, were searched utilizing key terms. PRISMA systematic search procedures identified 44 studies meeting predetermined inclusion criteria. Participant characteristics, the type of physical activity performed, the nature of the physical activity program/delivery, anxiety-related outcomes, and research methodology was evaluated for each study. Each paper included was appraised and scored for risk of bias using Cochrane Handbook for Systematic Reviews of Interventions risk of bias tool. Titles and abstracts of 44 articles were reviewed and 8 articles met inclusion criteria which evaluated interventions. Evidence from 8 studies suggests that yoga, a community-based football program, an app-assisted walking program, group exercise programs, and horseback riding interventions reduced anxiety for autistic people. The studies included in this systematic review provide strong-to-moderate evidence that physical activity can reduce anxiety for autistic children and adults. However, additional research is needed to identify which mode of physical activity is most beneficial for anxiety reduction. Further, future research should evaluate frequency, duration, and intensity and their effects on anxiety for autistic people.

## Introduction

In 2023, the Centers for Disease Control and Prevention (CDC) reported 1 in 36 children and 1 in 45 adults in the United States are diagnosed with autism (CDC, 2023). Autism is a neurodevelopmental disorder characterized by persistent deficits in social interaction and communication, as wells as restricted, repetitive patterns of behavior, activities, or interests (American Psychiatric Association, 2013). Autism affects people who are diagnosed differently; learning, thinking, and problem-solving abilities in individuals with autism can range from gifted abilities to severe deficits (CDC, 2023).

According to a caregiver-reported national survey, 40% of autistic children experience anxiety (Kerns et al., 2020), and 20% of autistic adults have been diagnosed with anxiety (Nimmo-Smith et al., 2020). A number of explanations have been developed to explain the increased levels of anxiety for autistic people. First, it is theorized that anxiety may be due to an aversion to situational change or experiencing unfamiliar environments (Gillott et al., 2001; White et al., 2009). Autistic individuals may have difficulty processing unfamiliar and changing environments that lead to feelings of uncertainty (Gillott et al., 2001). Gillott et al. (2001) suggests that stereotypical behaviors like twirling, rocking, or hand flapping, and complex behaviors like repetitive questioning are triggered by anxiety for autistic people. These behaviors are considered a response by the individual to attempt to calm themselves in response to unfamiliar environments (Gillott et al., 2001).

Another proposed theory to describe the relationship between anxiety and autism is the individual’s self-awareness (Gillott et al., 2001). Anxiety in autistic individuals may be mediated by one’s level of self-awareness or self-perceived disability (Gillott et al., 2001). Many autistic people are aware of their difficulties understanding situations and environments and their social disconnectedness (White et al., 2009). The awareness of the impact of autism on their life may be associated with increased anxiety and exacerbate social isolation, awkward social interaction, and avoidance of social situations (Gillott et al., 2001; White et al., 2009). Increased social isolation may also be due to some autistic people’s fear of being inaccurately perceived by the general population, this fear caused by people’s innate desire to be accepted by others (Mitchell et al., 2021). Additionally, the general population may not be able understand autistic individuals due to lack of insight into the culture and minds of autistic people, the combination of these misunderstandings and fears has been referred to as the ‘double empathy’ problem (Mitchell et al., 2021). Autistic people have identified belonging to be a critical component of overall well-being, and this fear of being misunderstood or misperceived combined with the general population’s lack of insight can lead to a detrimental impact on mental health (Mitchell et al., 2021). Behavioral manifestations of anxiety, self-perceived disability, and the double empathy problem may lead to increasing social isolation, creating a cycle of progressively negative effects on the individual’s mental and social health (Gillott et al., 2001; Mitchell et al., 2021; White et al., 2009). Due to the prevalence of anxiety for autistic people and its adverse effects, there is a critical need to develop effective interventions which address anxiety symptoms for autistic people and interrupt the anxiety cycle.

In the general population, two common methods for managing anxiety include medication and psychotherapy. Both methods can produce positive outcomes, such as potentially aiding in the relief of anxiety symptoms, but they also have some notable drawbacks. Medications and therapy can be expensive and difficult to access. Additionally, medication may cause unwanted side effects, including poor concentration, motor incoordination, drowsiness, mental confusion, vertigo, and muscle weakness, which can negatively influence mental health or daily function (Longo & Johnson, 2000). Also, nearly one third of patients do not respond to medication, therapy, or the combination of both (Kandola et al., 2018).

Physical activity has been shown to lower anxiety in the general population by chemically altering the brain, decreasing anxiety sensitivity, increasing self-efficacy, and improving quality of life (DeBoer et al., 2012). Physical activity can aid with regulation of the stress response via the hypothalamic–adrenal axis or glucocorticoid circulation, and it stimulates a broad range of neurogenic processes which promote proper brain functioning, particularly in brain regions that are associated with anxiety and stress (Kandola et al., 2018). According to a study by Broman-Fulks et al. (2004), both high-intensity physical activity and low-intensity physical activity was noted to decrease anxiety sensitivity in the general population. Anxiety sensitivity often causes a person to misinterpret and catastrophize physiological sensations, which can lead to a sense of panic (Broman-Fulks et al., 2004). Physical activity mimics some of the physiological symptoms associated with anxiety such as rapid breathing, sweating, and increased heartbeat, and it is believed that exposure to these physical sensations in a positive, healthy environment can be associated with lower anxiety sensitivity over time (Broman-Fulks et al., 2004). Physical activity can improve self-efficacy and elevate mood by increasing feelings of mastery after completing a goal task (Mikkelsen et al., 2017). Physical activity has been shown to improve quality of life in the general population by supporting weight management, reducing depression and anxiety, promoting better sleep, reducing the risk for more than 25 chronic medical conditions, improving cognitive performance, and improving academic performance (Erickson et al., 2019; Kandola et al., 2018; Warburton & Bredin, 2017).

If physical activity is an affordable and accessible alternative for managing anxiety for the general population, we should investigate if autistic individuals gain the same benefits from physical activity (Broman-Fulks et al., 2004). Intervention strategies designed for autistic individuals who experience anxiety are lacking, and current intervention methods are most often limited to cognitive behavior therapy, self-calming techniques from professionals, and pharmaceutical management (Adams et al., 2019; Trembath et al., 2012). Given the prevalence of anxiety in autistic individuals, there is a need for evidence-based, accessible, and cost-effective interventions to manage anxiety and related symptoms. Therefore, the aim of this systematic review is to review the literature describing the impact physical activity on anxiety symptoms for autistic individuals.

## Methods

This systematic review focused on what is the effectiveness of physical activity interventions for relieving anxiety symptoms for autistic people. We utilized Preferred Reporting Items for Systematic Reviews and Meta-Analyses (PRISMA) to guide our methodology (Page et al., 2021). First, we identified our PICO (population, intervention, comparison, outcome) question (Yamada et al., 2019). Then, we performed an exhaustive search on three databases, PsychInfo, Cochrane RCTs, and PUBMED. After the search was completed, we collected and assessed over 1000 articles. The protocol for this systematic review was not registered.

### Inclusion and Exclusion Criteria

The search inclusion criteria were as follows: any publish year, any age for participants, any design involving a physical activity intervention, any setting involving physical activity, and any duration of follow up. The studies extracted from the databases (e.g., PsychInfo, Cochrane RCTs, and PUBMED) must meet the criteria set by the PICO question to be included in the review. The population is autistic people, the intervention is physical activity, there is no requirement for a comparison group, and the outcomes are measures of anxiety. The exclusion criteria were if a study did not have the previously stated population, intervention, or outcome.

### Search Procedures

**Fig. 1:**
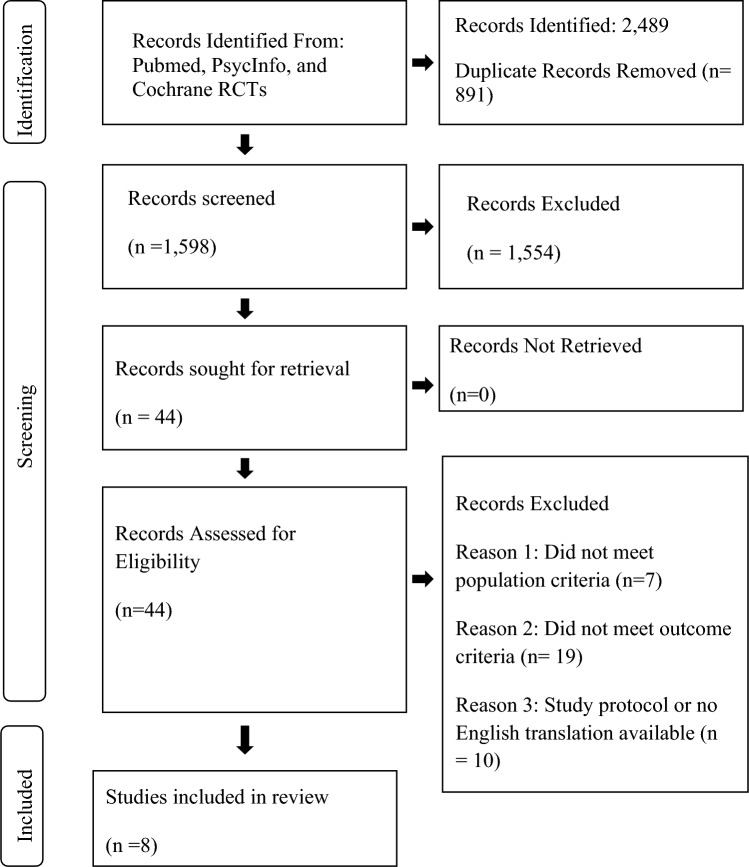
Identification of studies via databases

The primary investigator conducted a search in three databases during March of 2023, PsychInfo, Cochrane RCTs, and PUBMED. The search was limited to studies with human subjects and peer-reviewed journals, but publication year was not restricted. We utilized the following “population” key words to search all databases: autism, autism spectrum disorder, developmental disability, “intervention” key words: physical activity, regimen, physical task, training, workout, movement, performance, gym, aerobic, strength, lifting, walking, running, cycling, swimming, and “outcome” key words anxiety symptoms, anxiety sensitivity, anxiety, mood, mood disorders, mental health, anxiety disorder, generalized anxiety disorder, post-traumatic stress disorder, PTSD, separation anxiety, emotions, temperament, and cortisol. We used the advanced search option on the databases to utilize the ‘AND’ feature and search all the combinations of these words, three at a time. The combination would be a “population” key word, an “intervention” key word, and an “outcome” key word (“autism” AND “training” AND “anxiety”). This process was replicated for each of the three databases, PsychInfo, Cochrane RCTs, and PUBMED. We found 2489 studies. After removing duplicates, 1598 studies remained (Fig. 1). Two members of the research team read the abstracts and the titles of all 1598 papers to determine if they met the inclusion and exclusion criteria (Fig. 1).

### Record Selection

The following PRISMA criteria were followed: screen based on titles/abstract, multiple, independent reviewers, and report how disagreements were resolved (Brennan & Munn, 2021). After removing duplicates, 1598 papers were included and assessed for the inclusion/exclusion criteria by reading the title and the abstracts by two authors, independently. Conflicts were resolved by a third author, and a consensus discussion of all three reviewers yielded 44 records for full text review. The 44 records underwent full text review using the same inclusion and exclusion criteria as stated in section “Inclusion and Exclusion Criteria”. After reviewing the 44 records, 8 studies met the inclusion and exclusion criteria and are included in this review. After full text review, 36 records were eliminated because they did not include the correct population or outcome measures, they were not a study (book, treatment protocol), or an English version of the article was not available. Eight studies met the inclusion and exclusion criteria and were included in the analysis.

### Quality Assessment

The data extracted from the articles by the primary investigator for this systematic review included the authors of the articles, the year of publication of the articles, the levels of evidence, the number of participants, the participants’ ages, the sex of the participants (if reported), inclusion criteria (if reported), exclusion criteria (if reported), intervention procedures, control group procedures (if included), outcome measures, and results. After the primary investigator organized the data for extraction into an evidence table (Table 1), the studies were evaluated for quality, and then evaluated for risk of bias. The quality of evidence for each study was evaluated by examining the levels of evidence for each article using Sackett’s levels of evidence (Sackett, 1989):

- Level I: Systematic reviews, meta-analyses, randomized controlled trials (RCT).
- Level II: Two groups, nonrandomized studies (e.g., cohort, case–control).
- Level III: One group, nonrandomized (e.g., before and after, pretest and posttest).
- Level IV: Descriptive studies that include analysis of outcomes (single-subject design, case series).
- Level V: Case reports and expert opinion that include narrative literature reviews and consensus statements.

**Table 1:** Evidence table for the physical activity intervention studies and their effects on anxiety for autistic children and adults

Author/year	Level of evidence/participants/inclusion and exclusion criteria	Intervention and control groups	Outcome measures	Results
Carey et al. (2022)	Level III *n* = 24 Inclusion criteria: ages 5–18, diagnosis of ASD by psychologist, moderate to severe ASD—measured by the Gilliam Autism Rating Scale—2nd Edition (GARS). The authors defined moderate ASD as a score of 10–33 on the GARs, and severe ASD as a score of more than 34 on the GARS Exclusion criteria: no diagnosis of ASD, if potential participants had any medical conditions or injuries that prevented them from participating in exercise, and if a guardian or doctor advised they should not participate in exercise	Intervention: all participants participated in a 16-week exercise program intervention. The sessions occurred 3 days a week for 1 h, and included a warm-up, main activities, and stretching at the end. The main activities focused on developing and progressing fundamental motor skills like catching, throwing, kicking, balancing, dodging, striking, and jumping. The main activities also included some group games, involving all of the children	The ASC-ASD was completed by teachers, parents, or both to measure participants’ anxiety levels in home and school settings	According to the ACS-ASD reported by teachers, there was a significant decrease in performance anxiety, anxious arousal, uncertainty, and total ACS-ASD scores when comparing baseline to the final session (week 16) (*p* < 0.001). According to the ACS-ASD reported by parents, there was no significant decrease in total ACS-ASD scores when comparing baseline to the final session (week 16)
Gehricke et al. (2022)	Level II *n* = 53—physical activity group *n* = 64—sedentary activity group Inclusion criteria: participants were between the ages of 6 and 12, ASD diagnosis, ability to participate in moderate exercise, ability to follow basic directions, and anxiety defined as the more than the 93rd percentile on the CBCL or a total score more than 25 on the SCARED Exclusion criteria: behaviors that prevented participation in group activities, any medical condition that would be adversely affected by moderate exercise, sensory impairments that could affect participation, and children who were actively participating in other community-based exercise programs or were planning to do so	Physical Activity group: 8 weeks—3 physical activity sessions a week for 40–50 min The physical activity program included 10 min of warm-up,10 min of moderate intensity aerobic exercises at 65–85% of maximum heart rate, then 5 min of bone and muscle strength activities using body weight only, then 5 min of moderate intensity aerobic exercise combined with muscle strength activities, and lastly, they completed 5 min of cool down activities Sedentary Activity Group: 8 weeks—3 Lego or Minecraft sessions a week for 45 min—participants got to decide if they would prefer to play with Legos or play Minecraft Lego sessions: first, a 5-min introduction to trained study staff and LEGO rules and planning which role the participant will begin with. After the roles were assigned, engineer would give descriptions of the pieces needed and directions on how to assemble the model, and the builder would follow the directions, collect the pieces, and put the pieces together. The roles then switched after 15 min, and they played in the other role for the next 15 min. After the participant had a chance to play in both roles, they spent 15 min cleaning Minecraft: first, a 5-min introduction to trained study staff and Minecraft rules for the participant, as well as planning participant’s goals for the session. Next, participants participated in 20 min of group play in daytime mode through the game. Then, they played 15 min of survival play in night mode. Finally, the last 5 min were used to discuss how the participant felt about the intervention	The CBCL DSM-5 anxiety subscale, the SCARED—self-reported, the parent-rated CSHQ, the PAQ-C, and salivary cortisol were collected at baseline, week 3, week 6, week 8, week 12 as a follow up, and week 16 as a second follow up	Both the physical activity and sedentary activity groups had significant improvements in anxiety at weeks 3, 6, and 8 as rated by the CBCL DSM-5 anxiety subscale, with larger improvements in the exercise intervention group (*p* < 0.001). The SCARED measure reported significant improvements for both groups at 8 weeks (*p* < 0.001), with larger improvement in the sedentary group. The CSHQ results concluded sleep quality was significantly improved for the physical activity group a week 8 (*p* = 0.003). The PAQ-C results showed a significant increase in the physical activity group at week 8 compared to the sedentary group (*p* = 0.003). There were no significant acute or chronic changes to salivary cortisol for both groups
Hillier et al. (2011)	Level III *n* = 18 Inclusion criteria: ages 13–27, diagnosis of ASD, considered to be “high-functioning” the authors define this as: not having significant communication impairments, the ability to follow multi-step instructions, and participants could not exhibit behavioral challenges like physical or verbal aggression, and a note from a physician that said they are able to participate in a physical activity program	Intervention: all participants completed an 8-week low level physical activity program. This program included 5 min of warm-up activities, 10 min of aerobics, 10 min of flexibility exercises, 10 min balance activities, 10 min of strengthening activities, 5 min of team activities, and 10 min of cool down activities. These sessions occurred once a week for 75 min	Salivary cortisol levels were measured through saliva samples gathered at the beginning and end of 3 sessions, weeks 2, 4, and 6 Participants self-reported anxiety levels before and after each physical activity session using STAI	There was a significant difference in salivary cortisol before and after each individual training session (*p* = 0.001), but not across the 3 weeks (*p* = 0.575) There was a significant difference in anxiety measured by the self-reported STAI before and after each individual training session (*p* = 0.002), but not across the 8 weeks (*p* = 0.365)
Howells et al. (2020)	Level II *N* = 40 Inclusion criteria: a pre-existing diagnosis of ASD under the DSM-5 guidelines and ages 5–12	Intervention: 19 children who were already participating in the program at local Auskick clubs. The group physical activity sessions included practicing fundamental motor skills and football related skills 60–90 min once a week, minimum of 5 sessions Control group: 21 children not enrolled in the football program, or have been engaging in more than 30 min a week of organized physical activity for the duration of the study. The control group were permitted to engage in their typical, everyday routines	Parents completed the CBCL which included the DSM-Oriented Anxiety and Depressive Problems and the Vineland-3	Significant decreases in total problem behaviors (*p* = 0.048), social problems (*p* = 0.01), and internalizing problems compared to the control group (*p* = 0.01) as measured by the CBCL were reported post-intervention for the intervention group. DSM-oriented anxiety problems for the intervention group showed a significant decrease in scores post-intervention compared to the control group (*p* = 0.001). No significant results were reported by the Vineland-3. No significant differences reported in the control group
Keino et al. (2009)	Level III *n* = 4 No inclusion nor exclusion criteria listed	Intervention: 4 children participated in Pyscho-Educational Horseback Riding Program (PEHR) Provides, which carefully planned psychological and educational care for each child while they are riding horseback. These riding sessions progressed with time and eventually included the participants playing psycho-educational games, experiencing the horse both walk and trot, and ended with the participants doing their favorite activities. The children participated in PEHR anywhere from 1 to 3 years	Evaluation by the mother of the participant with the HEIM	A statistical difference was measured baseline values and another time point for part 8 of the HEIM scale Part 8: fear or nervousness’ (degree of crying or being afraid) (*p* < 0.05) 1. Acts very anxious and cries very hard for a long time 2. Cries at first stops after a while 3. Face shows great anxiety 4. Face shows slight anxiety As well as a statistical difference post intervention for the measures of human relationship, imitation, emotional expression, adaptation to change, visual response, and verbal communication subscales of the HEIM
Lee et al. (2022)	Level II *n* = 20 Inclusion criteria: 18 years of age or older, diagnosed with ASD, medical diagnosis of anxiety or self-identifying as experiencing anxiety in the past 3 months or more, no previous experience of using the physical activity mobile apps that are used in this study, and cognitive ability to understand the purpose of the study Exclusion criteria: low cognitive function, people with co-occurring intellectual disabilities, or mobility impairments	Intervention: participants were randomly assigned to the PuzzleWalk or Google Fit group. Both are applications designed to promote physical activity. The intervention lasted 8 weeks, however after collecting baseline data and distributing materials for the data collection, data collection occurred again during the 4th week to reduce the novelty effect in response to the accelerometer and the applications. Data were collected during the 4th and 8th weeks	Accelerometer data was collected with a waist accelerometer. A self-report survey was used to assess the use of the physical activity applications during the 4th week and at the start and at the end of the 8th week. The BAI—self-report was completed at baseline (week 1), during the 4th week, at the start of the 8th week, and at the end of the 8th week. Also, daily during 1st, 4th, and 8th weeks, time-specific and type of anxiety trigger questions were asked via an app developed by the research team to better identify the contexts of potential anxiety triggers such as environmental, psychological, or sensory factors	There was a significant difference for pre- and post-testing was in daily steps for both groups (*p* = 0.04). Both groups had a decrease in the BAI scored from pre-test to post-test, although these were not significant
Peters et al. (2021)	Level I RCT *n* = 20 Inclusion: age 6–13 years old, diagnosed with ASD by a community provider, score greater than a 15 on the Social Communication Questionnaire (SCQ), meet clinical cut-offs for ASD on the Autism Diagnostic Observation Schedule (ADOS), Autism Diagnostic Observation Schedule, second edition (ADOS-2), or the Social Responsiveness Scale, Second Edition (SRS-2), combined score more than 25 on the irritability and hyperactivity subscales of the ABC-C, score more than 55 on the Leiter International Performance Scale, Third Edition (Leiter-3), can participate in 10-min of riding while following safety rules, meets PATH Intl physical, mental, and emotional standards Exclusion criteria: weigh more than 200 pounds and participated in equine-assisted services for 2 h or more in the last 6 months	Intervention Group: 11 children with ASD. 10 weeks of Occupational Therapy in an Equine Environment: harnessing Occupation to Address Self-Regulation, Social Communication and Play and in Youth with Autism (OT^ee^ HORSPLAY) 60-min sessions once a week that included greetings, activities with the horses, goodbye, and caregiver debrief. The activities were individualized for the participants’ movement goals Control Group: 9 children with ASD, received 10 weeks of didactic training and modeling led by OT graduate students, and the OT^ee^ intervention after the first group completed it and data was collected	GAS, completed by an occupational therapist during a semi-structured interview with parents and participants, the irritability and hyperactivity subscales of the ABCC—parent reported, SRS-2—parent reported, PEDI-CAT ASD, parents completed a survey that included three open-ended questions about the best and worst aspects of the intervention or their child, and hair cortisol content (HCC) of the participants was collected at baseline and after the intervention was completed	The intervention group reported significant improvements in goal performance as rated by the GAS (*p* < 0.001), and social motivation as rated by the SRS-2 (*p* = 0.033), and significantly reduced irritability as measured by the ABC-C (*p* = 0.04) after the intervention. There were no other reported significant changes for the intervention group. The control group also had significant improvements in goal performance as rated by the GAS (*p* = 0.002), and social motivation as rated by the SRS-2 (*p* < 0.001), and no other significant results were reported. Also, the HCC resulted in non-significant increases after the intervention
Tanksale et al. (2021)	Level I RCT *n* = 61 children with ASD and their parents Inclusion criteria: being between the ages 8 and 12 and a verified ASD diagnosis Exclusion criteria: a non-verified diagnosis of ASD and no fluent speech or non-verbal	Intervention: a yoga-based program that occurred once a week for 1 h for 6 weeks. The program included breath-centered postures for the first part, for the second part the children learned mindfulness through games and activities, and the parents attended an education debriefing session that focused on barriers and how to facilitate home practice Control group: waitlist controls—could receive intervention after if they so choose	The BRIEF—parent questionnaire, CSHQ—parent reported, the ASC-ASD—parent version, GAS—parent report, the EAQ—children reported, and the ASC-ASD—self-reported by the children	According to the post-intervention results for the CSQ there was a significant difference between groups for bedtime resistance (*p* = 0.023), sleep onset delay (*p* = 0.024), sleep disordered breathing (*p* = 0.05) and sleep anxiety (*p* = 0.015). According to the EAQ, there was significant between group differences post-intervention for verbal sharing of emotions (*p* = 0.005) and analysis of emotions or willingness to understand one’s emotions (*p* = 0.047). According to the ASC-ASD—self-reported by the children, there was a significant difference for performance anxiety at the follow-up data collection for the intervention group, compared to the control group (*p* = 0.028)

We have included an evidence table (Table 1) that identifies the authors, the year, level of evidence, participants, inclusion criteria, intervention and control groups, outcome measures, and results. We evaluated the risk of bias using a format adapted from the Cochrane Handbook for Systematic Reviews of Interventions (Green & Higgins, 2023) and the data from that assessment is included in Table 2. In this table the grading criteria were as follows: low risk of bias (+), unclear risk of bias (?), high risk of bias (−) (Table 2). Low risk of bias means the study included a valid approach for allocation, a low dropout rate, actively attempted to prevent bias in measure outcomes and reporting (Shaw et al., 2013). Unclear risk of bias means the study could be susceptible to some bias but not enough to invalidate the results, these may not include appropriate allocation techniques, high drop-out rates, or biases in reporting (Shaw et al., 2013). Finally, high risk of bias means the study may have significant bias that could invalidate the results, this may be due to a combination of lack of allocation techniques, no means to prevent bias, and a high drop-out rate (Shaw et al., 2013). Two authors reviewed the quality of the evidence and the risk of bias independently. Then, the authors discussed their results, and there were no disagreements to resolve. After the primary author gathered and organized the data into the evidence table, each author reviewed the evidence table for accuracy.

**Table 2:** Risk of bias table

Citation	Selection bias	Performance bias		Detection bias		Attrition bias	Reporting bias
	Random sequence generation	Allocation concealment	Blinding of participants and personnel	Blinding of outcome assessment: self-reported outcomes	Blinding of outcome assessment: objective outcomes	Incomplete outcome data	Selective reporting
Carey et al. (2022)	−	−	−	+	+	+	+
Gehricke et al. (2022)	+	−	−	+	+	+	+
Hillier et al. (2011)	−	−	−	−	+	+	+
Howells et al. (2020)	+	+	−	−	−	+	+
Keino et al. (2009)	−	−	−	−	+	+	+
Lee et al. (2022)	+	−	−	−	+	+	+
Peters et al. (2021)	−	−	−	−	−	+	+
Tanksale et al. (2021)	+	−	−	−	+	+	+

Note: Categories for risk of bias are as follows: Low risk of bias (+), unclear risk of bias (?), high risk of bias (−)

## Results

The original search of the three databases in March of 2023, resulted in 2489 articles for review. After removing duplicates, 1598 studies remained. Two members of the research team reviewed all abstracts and interrater reliability was moderate agreement (*k* = 0.66) (McHugh, 2012). The original two reviewers met with a third reviewer to discuss disagreements, 58 total disagreements. Finally, the abstracts that were agreed upon by the original reviewers, as well as the articles the third reviewer chose to include had their full-text reviewed. The number of full-text manuscripts reviewed was 44. Figure 1 presents the PRISMA flow chart for study selection. The eight records that met the criteria for inclusion and are included in this review included two level 1 RCT, three level II non-randomized studies with two groups, and three level III one group non-randomized studies. These studies also included a range of anxiety measures including salivary cortisol levels, the State-Trait Anxiety Inventory (STAI), the Anxiety Scale for Children-ASD (ASC-ASD), the Screen for Child Anxiety Related Disorders (SCARED), the Aberrant Behavior Checklist-Community (ABCC), hair cortisol content (HCC), the Child Behavior Checklist (CBCL) with the Diagnostic Statistical Manual of Mental Disorders-5 (DSM-5) anxiety subscale, the Human–Equips-Interaction on Mental activity scale (HEIM), and the Beck Anxiety Inventory (BAI) (Carey et al., 2022; Gehricke et al., 2022; Hillier et al., 2011; Howells et al., 2020; Keino et al., 2009; Lee et al., 2022; Peters et al., 2021; Tanksale et al., 2021). Results from these studies suggest there is strong evidence that engagement in community organized football programs (Howells et al., 2020; Level II) and yoga programs (Tanksale et al., 2021; Level I RCT) reduces anxiety in autistic children. We conclude that this is strong evidence based on the levels of evidence included, Level II and a Level I. In addition, there is moderate evidence that motor interventions involving horseback riding reduces anxiety in autistic children (Keino et al., 2009; Level III; Peters et al., 2021, Level I RCT). We conclude that this is moderate evidence based on the levels of evidence included, Level III and a Level I. Also, there is moderate evidence to support the use of general physical activity programs to reduce anxiety of autistic adults and children (Carey et al., 2022, Level III; Gehricke et al., 2022; Level II; Hillier et al., 2011, Level III; Lee et al., 2022, Level II). We conclude that this is moderate evidence based on the levels of evidence included, two Level IIIs and two Level IIs. The total sample considered in the resulting studies included 304 total participants, 47 females, 145 males, and gender for 130 participants were not reported (Table 3). Additional sample characteristics are outlined in Table 3.

**Table 3:** Sample characteristics for included studies

Author/year	Total number of participants	Ages	Diagnosis	Gender	Co-occurring conditions	Race/ethnicity
Carey et al. (2022)	24	5–18	Diagnosis of ASD by psychologist Moderate to severe ASD—the authors defined moderate ASD as a score of 10–33 on the GARs, and severe ASD as a score of more than 34 on the GARS	Males = 24 Females = 0	Not reported	Not reported
Gehricke et al. (2022)	117	6–12	Diagnosis of ASD Anxiety defined as the more than the 93rd percentile on the CBCL or a total score more than 25 on the SCARED	Not reported	Not reported	Not reported
Hillier et al. (2011)	18	13–27	Diagnosis of ASD Considered to be “high-functioning” the authors define this as: not having significant communication impairments, the ability to follow multi-step instructions, and the absences of behavioral challenges like physical or verbal aggression	Males = 16 Females = 2	Not reported	Not reported
Howells et al. (2020)	40	5–12	diagnosis of ASD under the DSM-5 guidelines	Males = 37 Females = 3	ADHD diagnosis (*n* = 14) Anxiety or Depression diagnosis (*n* = 11) Vision/hearing impairment (*n* = 7)	Not reported
Keino et al. (2009)	4	7–17	Autism (*n* = 2) Pervasive developmental disorders (*n* = 2)	Males = 4	Not reported	Not reported
Lee et al. (2022)	20	18+	Diagnosed with ASD, medical diagnosis of anxiety or self-identifying as experiencing anxiety in the past 3 months or more	Males = 9 Females = 15	Not reported	Not reported
Peters et al. (2021)	20	6–13	Diagnosed with ASD by a community provider, score greater than a 15 on the SCQ, meet clinical cut-offs for ASD on the ADOS, ADOS-2, or SRS-2, combined score more than 25 on the irritability and hyperactivity subscales of the ABC-C, score more than 55 on the Leiter-3	Males = 16 Females = 5	ADD/ADHD diagnosis (*n* = 9) OCD diagnosis (*n* = 1)	Latino/Hispanic (*n* = 5) Asian (*n* = 1) Black (*n* = 1) White (*n* = 15) Multi-racial (*n* = 4)
Tanksale et al. (2021)	61	8–12	Diagnosed with ASD	Males = 39 Females = 22	ADHD diagnosis (*n* = 22) Diagnosed anxiety disorder (*n* = 16) Parent reported anxiety (*n* = 59) Parent-reported sleep problems (*n* = 40)	Not reported

There is strong evidence suggesting the structured, community organized physical activity program reduces anxiety for autistic individuals (Howells et al., 2020; Tanksale et al., 2021). Howells et al. (2020) performed a study that included 40 autistic children. Nineteen autistic children were assigned to the intervention group because they were participating in a football program at local Auskick clubs, and 21 autistic children were in the comparison group (Howells et al., 2020). Though not involved in the football program, those in the comparison group could participate in their typical routines (Howells et al., 2020). The football program included sessions where children were given the opportunity to practice fundamental motor and football related skills in a group setting (Howells et al., 2020). These sessions occurred for 60–90 min once a week, and participants completed a minimum of 5 sessions (Howells et al., 2020). Parents completed the CBCL which included the DSM-Oriented Anxiety and Depressive Problems, and social functioning and communication were assessed through the Vineland Adaptive Behavior Scale-third edition (Vineland-3) (Howells et al., 2020). Decreases in total problem behaviors, social problems, and internalizing problems as measured by the CBCL were reported post-intervention for the intervention group compared to the control group (Howells et al., 2020). Also, DSM-oriented anxiety problems for the intervention group showed a significant decrease in scores post-intervention compared to the control group (Howells et al., 2020).

One study utilized yoga as an intervention. Tanksale et al. (2021) recruited 61 autistic children, aged 8–12 years, and their parents, 31 completed the intervention and 30 were assigned to the control group and placed on a waitlist for the intervention. The program took place once a week, for 1 h, for 6 weeks,  and included breath-centered postures that both the parents and children took part in, games and activities that promoted mindfulness for the children, and parents attended an educational session on how to facilitate home practice (Tanksale et al., 2021). The primary outcomes were measured pre-intervention, post-intervention, and at follow-up (Tanksale et al., 2021). Measures included the parent reported Behavior Rating Inventory of Executive Function (BRIEF), Children’s Sleep Habits Questionnaire (CSHQ), the ASC-ASD parent report, Goal Attainment Scale (GAS), and the child reported Emotion Awareness Questionnaire (EAQ), and the ASC-ASD self-report (Tanksale et al., 2021). According to the post-intervention results for the BRIEF significant group differences were reported for the global executive composite, cognitive regulation index, behavior regulation index, inhibit, and organization of material subscales, suggesting improvements in executive function for the intervention group (Tanksale et al., 2021). According to the post-intervention results for the CSHQ there was a significant difference between groups for bedtime resistance, sleep onset delay, and sleep anxiety, showing improved sleeping habits for children post-intervention (Tanksale et al., 2021). According to the EAQ, there was significant improvement post-intervention for verbal sharing of emotions and analysis of emotions or willingness to understand one’s emotions for the intervention group compared to the control group (Tanksale et al., 2021). According to ASC-ASD—self-report, there was a significant decrease for performance anxiety at the follow-up data collection for the intervention group compared to the control group (Tanksale et al., 2021). There were no other significant results reported (Tanksale et al., 2021).

Moderate evidence supports motor interventions involving horseback riding as a means to reduce anxiety in autistic children (Keino et al., 2009; Peters et al., 2021). Keino et al. (2009) performed a study with a Pyscho-Educational Horseback Riding Program (PEHR) intervention and included two autistic children and two children with pervasive developmental disorder. The PEHR program provides individualized planned psychological and educational care for each child while they are riding horseback, riding the horse on paths with no other conditions, psycho-educational games on the horse, and behavioral and psychological tasks on the horse (Keino et al., 2009). They used the Human–Equips-Interaction on Mental activity (HEIM) scale, a behavioral scale for evaluating the effect of human–equine relationships on mental activity (Keino et al., 2009). They reported a significant difference post-intervention for part eight of the HEIM scale which rated fear or nervousness as the child was presented with the horse they were going to ride. Each child decreased their level of anxiety when comparing their results from pre- and post-intervention as observed and graded by the investigators (Keino et al., 2009). Also, there were statistical differences reported post intervention for the measures of human relationship, imitation, emotional expression, adaptation to change, visual response, and verbal communication subscales of the HEIM (Keino et al., 2009). No significant differences were noted in the sudden movement, fixation behavior, and nonverbal communication subscales of the HEIM (Keino et al., 2009). Another study included a 10-week occupational therapy intervention program in an equine environment called OT^ee^ HORSPLAY (Peters et al., 2021). This study included 20 autistic children, randomized into either the OT^ee^ HORSPLAY group, which included 10 weeks of occupational therapy in an equine environment once a week for 60 min, or the waitlist control group, which participated in 10 weeks of didactic training and modeling led by OT graduate students. The control group also completed the OT^ee^ HORSPLAY intervention after the first group completed it and data were collected (Peters et al., 2021). Outcome measures included the GAS, Pediatric Evaluation of Disability Inventory Computer Adaptive Test, Autism Spectrum Disorder Module (PEDI-CAT ASD), HCC, the irritability and hyperactivity subscales of the ABC-C, the Social Responsiveness Scale Second Edition (SRS-2), and a survey for the parents that included three open-ended questions about the best and worst aspects of OT^ee^ HORSPLAY for their child. Peters et al. (2021) reported improvements in goal performance progress, occupational performance goals, social motivation for the intervention group. They also report significantly reduced irritability for the intervention group (Peters et al., 2021). No other significant results were reported post-intervention for the OT^ee^ HORSPLAY group (Peters et al., 2021).

Several studies included general physical activity programs as their intervention and provide moderate support for this intervention in anxiety reduction for autistic individuals (Carey et al., 2022; Gehricke et al., 2022; Hillier et al., 2011; Lee et al., 2022). One study included an 8-week physical activity program that included aerobics, flexibility, balance, strength training, and team activities was used as an intervention for 18 autistic young adults, ages 13–27 (Hillier et al., 2011). This study analyzed the effects of the intervention on stress and anxiety for autistic young adults, therefore they measured salivary cortisol levels as well as participant-reported STAI (Hillier et al., 2011). They reported significant reductions in salivary cortisol levels before and after each training session, but not across the 3 weeks, from week 4 to week 6 or week 2 to week 4 (Hillier et al., 2011). Also, there was a significant reduction in self-reported anxiety before and after each training session, but not a significant reduction in self-reported anxiety across the 8-weeks, as reported by the STAI (Hillier et al., 2011). A study by Carey et al. (2022) included a 16-week exercise program intervention, the sessions occurred three times a week for 1 h and included activities focused on developing and progressing fundamental motor skills like balancing, jumping, and catching, and group games. Participants level of anxiety was measured by the ASC-ASD, completed by parents, teachers, or both to evaluate the participant’s anxiety both in the home and school setting (Carey et al., 2022). According to the ACS-ASD reported by teachers, there was a significant decrease in total ACS-ASD scores when comparing baseline to the final session, but there was no significant decrease in total ACS-ASD scores reported by parents when comparing baseline to the final session (Carey et al., 2022). Another study by Gehricke et al. (2022) compared two groups of autistic children, one group of 53 completed 8 weeks of three physical activity sessions a week for around 50 min, and the other group of 64 completed 8 weeks of 3 LEGO or Minecraft sessions a week for 45 min. For each group the CBCL with the DSM-5 anxiety subscale, SCARED, the parent-rated CSHQ, Physical Activity Questionnaire for Older Children (PAQ-C), and salivary cortisol were collected at baseline, week 3, week 6, week 8, and a follow-up at week 16 (Gehricke et al., 2022). Both the physical activity and sedentary activity groups had significant improvements in anxiety at weeks 3, 6, and 8 as rated by the CBCL with the DSM-5 anxiety subscale, and the exercise intervention group had nominally larger improvements than the sedentary group (Gehricke et al., 2022). At 8 weeks, the SCARED measure reported significant improvements for both groups with a larger improvement in the sedentary group (Gehricke et al., 2022). According to the CSHQ results, sleep quality was significantly improved for the physical activity group, but not the sedentary group (Gehricke et al., 2022). Also, there were no significant chronic or acute changes to salivary cortisol for both groups (Gehricke et al., 2022). Lee et al. (2022) performed an app-assisted walking intervention for 24 autistic adults. Participants were randomly assigned to using the Puzzlewalk app or Google Fit app group, both are apps that use visual aids and goals to promote physical activity (Lee et al., 2022). The intervention occurred for 8 weeks, but data was collected during the fourth and eighth week to reduce the novelty effect of the apps (Lee et al., 2022). Accelerometer data, a self-reported BAI, and a questionnaire that assessed the usefulness of the applications for participants were collected from the participants (Lee et al., 2022). Anxiety levels were not significantly reduced during this intervention, but results suggest that these applications are effective at promoting physical activity (Lee et al., 2022).

## Discussion

The results of the systematic review provide evidence of the use of physical activity to address anxiety often observed in autistic individuals. The evidence provides support for the use of foundational and structured group sport activities, yoga programs, horseback riding, and general physical activity programs to reduce anxiety in autistic individuals that often results in avoidance of social interaction, isolation, and increased internalization of problems (Carey et al., 2022; Gehricke et al., 2022; Gillott et al., 2001; Hillier et al., 2011; Howells et al., 2020; Keino et al., 2009; Lee et al., 2022; Peters et al., 2021; Tanksale et al., 2021; White et al., 2009).

According to the evidence examined in this review, there is strong evidence to suggest that participation in yoga programs and community-based football programs reduce anxiety in autistic children (Howells et al., 2020; Tanksale et al., 2021). Howells et al. (2020) compared anxiety levels between the intervention group, those participating in their local Auskick program, and a control group, and the intervention group showed a significant decrease in anxiety post-intervention compared to the control group (Howells et al., 2020). Group sport activity has been shown to decrease depressive symptoms, improve social abilities, and increase self-esteem in the general population (Pluhar et al., 2019). Though more research is needed, evidence suggest the effects of group sport may be a mediator of anxiety for autistic individuals. Tanksale et al. (2021) included a yoga intervention for autistic children and reported a significant decrease in performance anxiety and sleep anxiety for the intervention group (Tanksale et al., 2021). Yoga has been shown to improve motor skills, imitation skills, happiness ratings, self-control, and motor skills for autistic people (Hourston & Atchley, 2017; Kaur & Bhat, 2019; Shanker & Pradhan, 2022). These benefits combined with the potential reduction in anxiety for autistic people promote the need for further investigation into yoga interventions as an effective and beneficial intervention for autistic individuals.

According to the evidence included in this review, there is moderate evidence to suggest motor interventions involving horseback riding can reduce anxiety for autistic children (Keino et al., 2009; Peters et al., 2021). Horseback riding interventions focus on engaging an individual’s cognitive, sensory, and neuromotor systems and helping people achieve certain functional outcomes (Srinivasan et al., 2018). These interventions have been shown to be effective for autistic people for improving social and communication skills, self-esteem, and motor skills (Srinivasan et al., 2018). The results of this systematic review suggest that horseback riding interventions may have a mediating effect on anxiety as well.

There is moderate evidence that general physical activity programs, including app-assisted walking, aerobic exercise, muscle strength activities, balance activities, group games, catching and throwing activities, and flexibility training, can reduce anxiety for autistic adults and children (Carey et al., 2022; Gehricke et al., 2022; Hillier et al., 2011; Lee et al., 2022). In the general population physical activity can decrease anxiety symptoms by altering the chemicals within the brain, decreasing anxiety sensitivity, increasing self-efficacy, acting as a break from a stressful day, improving quality of life, decreasing psychological distress, and improving physical health (DeBoer et al., 2012; Kandola et al., 2018). Although there is limited evidence in the literature to suggest a mechanism for the reduction in anxiety due to physical activity for autistic people, we can hypothesize that any or a combination of the mechanisms in place for the general population also occur for autistic individuals.

The physical activity intervention implemented by Carey et al. (2022) was delivered on a school campus, included groups of 6–10 children at a time, and the educator had appropriate experience with delivering exercise program to autistic children. These criteria eliminate several barriers to physical activity identified through research like transportation, lack of others to participate in exercise with, and lack of appropriate support (Boucher et al., 2022; Hillier et al., 2020). These type of physical activity interventions provide a way to overcome these between-person barriers, and early interventions of such programs can help reduce within-person barriers like negative experiences with physical activity by providing a safe, enthusiastic, enjoyable experience with physical activity early for autistic people (Boucher et al., 2022). Also, it been reported that physical activity can improve motor skills for autistic people (Ruggeri et al., 2020), which is another barrier reported by parents of autistic children. Therefore, participation in a small group, appropriately supervised program located on a school campus could eliminate several barriers to participation, and potentially help autistic children experience the benefits of physical activity like a decrease in anxiety, improved motor skills, decrease stereotypical behaviors, and improved communication skills, all of with have been reported to be affected by physical activity (Bahrami et al., 2016; Howells et al., 2022; Huseyin, 2019; Keller et al., 2021; Must et al., 2015; Shanok et al., 2019; Zhao & Chen, 2018). Hillier et al. (2011) implemented a physical activity intervention for young autistic adults and autistic children, which resulted in significant changes in anxiety, and successfully recruited and retained 18 participants throughout the 8-week intervention. Although the methodology does not include attempts to overcome barriers in participation, they did recruit from a sample of people who were already participating in a physical activity program (Hillier et al., 2011). Future research could compare groups of those participating in physical activity programs and those who are not participating in physical activity programs to better understand why the barriers are overcome by some but not by others. App assisted physical activity interventions could serve as a way to overcome barriers like lack of physical activity enjoyment, by adding a gaming component, as well as lack of resources and transportation since it can be used almost anywhere, parks, neighborhoods, around a campus (Lee et al., 2022; Must et al., 2015). Overall, programs to increase physical activity participation for autistic people should focus on overcoming perceived barriers and improving feelings towards physical activity, so autistic people can receive the apparent of benefits of physical activity.

### Study Strengths and Limitations

This is the first systematic review, to our knowledge, of the influence of physical activity intervention on anxiety for autistic people. There was a limited number of studies that included our population, intervention, and outcome criteria. Six out of the eight studies included a population criterion of the participants being aged 18 and below, even though the average lifespan of autistic individuals is 40–58 years, depending on severity (Sala et al., 2020). Among these studies anxiety measurement methods varied, as well as who completed the measures, self-report, parent-report, investigator report, or teacher report. As outlined by Table 3, the studies included did not consistently report certain demographic information or sample characteristics. For example, only one study reported race/ethnicity. This is a limitation because this information could have provided insight into the applicability of the results to other people diagnosed with autism. Lastly, none of the studies included a community engagement component to provide autistic people an opportunity to help shape the intervention, or to solicit feedback on the intervention.

### Implications

The findings of this systematic review have the following implications:

- The evidence indicates that autistic people who experience anxiety benefit from participation in varied physical activity as a means to mediate anxiety and its effects.
- Autistic children and youth who experience anxiety should participate in general physical activity, specifically designed and structured physical activity, and/or sports on a regular basis.
- Physical activity opportunities should be made available to children and youth throughout the school day, including but not limited to teaching physical activities/physical games during recess, sports programs offered before and after school, and yoga activities in the classroom.
- Community programs should be developed to provide autistic children, youth, and adults with general physical activity and structured sports opportunities. Based on the results of this review these programs should focus on implementing programs involving group sport, yoga, motor interventions involving horseback riding, and/or general physical activity programs.
- Future research should focus on:
  - Narrowing intervention type, duration, and intensity, so we can provide the most effective recommendations for mediating anxiety symptoms through physical activity for autistic people.
  - Identifying the most reliable outcome measures for anxiety for autistic people.
  - Community-engaged projects in which participants contribute to the design of the intervention.
  - Increasing the range of age of participants included, so we can have a better understanding of the influence of physical activity on anxiety for autistic people during all stages of life (Table 3).

## Conclusion

Anxiety is a major concern for autistic people, it affects 40% of autistic children and 20% of autistic adults (Kerns et al., 2020; Nimmo-Smith et al., 2020). There is a potential for physical activity to be an effective, free intervention to relieve anxiety symptoms for autistic people. Physical activity as a means to reduce anxiety symptoms for the general population has been substantially investigated, however there is not as much research in this area for autistic people. This review compiled the literature currently available for this intervention, group, and outcome measures from three databases. We included a total of eight studies in this systematic review, and the results suggest there is moderate to strong evidence to support the use of different physical activity interventions for mediating anxiety symptoms for autistic children and adults.
